# Biology, Nesting Behavior, Genetic Diversity, and Conservation of Leatherback Sea Turtles: Insights From Thailand and Global Perspectives

**DOI:** 10.1002/ece3.71014

**Published:** 2025-02-20

**Authors:** Promporn Piboon, Janine Brown, Patcharaporn Kaewmong, Kongkiat Kittiwattanawong, Korakot Nganvongpanit

**Affiliations:** ^1^ Veterinary Medicine Chiang Mai University Chiang Mai Thailand; ^2^ Smithsonian Conservation Biology Institute Center for Species Survival Front Royal Virginia USA; ^3^ Phuket Marine Biological Center Phuket Thailand; ^4^ Department of Marine and Coastal Resources Ratthaprasasanabhakti Building (Building B) the Government Complex Bangkok Thailand

**Keywords:** conservation, *Dermochelys coriacea*, DNA, habitat, nesting behavior, population genetics

## Abstract

Sea turtles are large reptiles that inhabit the world's oceans and include seven extant species within two families: Cheloniidae and Dermochelyidae. These species are threatened globally, with several subpopulations listed as Endangered or Critically Endangered by the IUCN. Thailand hosts five sea turtle species, including leatherback turtles ( *Dermochelys coriacea* ), which are significant for their nesting sites along the Andaman Sea coast. Conservation efforts in Thailand include beach patrols, hatcheries, and community education to mitigate threats such as poaching and habitat destruction. Leatherback turtles, classified as “Vulnerable” by the IUCN and listed in CITES Appendix I, face challenges in estimating global population size due to their highly migratory nature. They are the largest sea turtles, with distinct physical characteristics such as leathery skin, lack of scales, a hard shell, and backward‐pointing spines in the throat that aid the passage of food. Leatherbacks reach sexual maturity at around 13–14 years of age and exhibit natal homing behavior for nesting. These turtles have low hatching rates; only 50% of eggs hatch, and just 2% of those hatchlings survive. Population genetic studies of leatherback turtles have been key to better understanding threats to survival, revealing low global mtDNA haplotype diversity, with notable recent radiation originating from the Indo‐Pacific region. Despite this low diversity, there is significant population structuring, which hints at hidden nesting populations and foraging grounds that may contribute to genetic variability. For that reason, relocating nests to favorable locations is one possible conservation measure. Other strategies must address habitat loss, pollution, bycatch, and climate change in protection efforts for this species, as well as ensuring global population connectivity to maintain the genetic diversity of these highly migratory turtles.

## Graphical abstract

Sea turtles, including the endangered leatherback, face threats from poaching, habitat loss, and climate change. In Thailand, conservation efforts focus on protecting nesting sites and improving hatchling survival. Leatherbacks, with low genetic diversity and high migratory patterns, require targeted measures to ensure their survival.

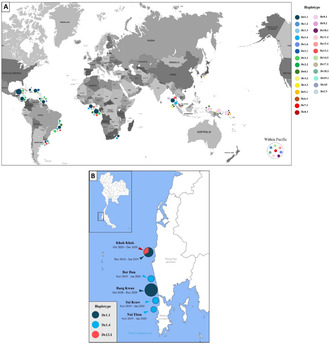

## 1 Introduction

Sea turtles are large, long‐lived reptiles inhabiting oceans around the world, with seven living species in the Order Testudines divided into two families: Cheloniidae and Dermochelyidae. Species include leatherback ( *Dermochelys coriacea* ), green ( *Chelonia mydas* ), loggerhead ( *Caretta* ), hawksbill ( *Eretmochelys imbricata* ), olive ridley ( *Lepidochelys olivacea* ), kemp's ridley ( *Lepidochelys kempii* ), and flatback ( *Natator depressus* ) sea turtles. Subpopulations of these species are listed as Vulnerable, Endangered, or Critically Endangered by the International Union for Conservation of Nature (IUCN) Red List of Threatened Species (Wallace et al. 2013).

Thailand is home to leatherback, green, loggerhead, hawksbill, and olive ridley turtles. Of these, the green and hawksbill are the most commonly observed in Thailand's coastal waters, while the leatherback is less common. Still, Thailand is important to leatherback sea turtle survival as it hosts important nesting sites for the species (west beach area in Phuket and Phang‐Nga). Efforts to protect leatherbacks in Thailand include initiatives such as beach patrols, hatcheries, and community education programs to reduce threats like poaching, habitat destruction, and accidental capture in fishing gear. Leatherback turtles ( *Dermochelys coriacea* ) are classified as “Vulnerable” by the International Union for Conservation of Nature (IUCN) and listed in the Convention on International Trade in Endangered Species (CITES) Appendix I (NOAA National Oceanic and Atmospheric Administration 2024). Estimating the global population size of leatherback sea turtles has been challenging because they are highly migratory, some traveling over 16,000 km from foraging to nesting grounds. Data from population genetic analyses have been used to estimate the population structure in nature, finding it is significant and that unidentified nesting populations and foraging grounds may contribute to genetic variability. This review describes the biology and distribution of leatherback turtles, including data on genetic diversity and population structure of the leatherback sea turtle.

## 2 Distribution and Biology of the Leatherback Turtle

### 2.1 Morphology and Unique Aspects

Among the seven species of sea turtles, the leatherback is the largest, with notably distinct morphology (Robinson and Paladino 2013). Body length, curved carapace length (CCL) averages around 130–190 cm for adults (Robinson and Paladino 2013) with weights between 275 and 560 kg for typical laying egg females (Georges and Fossette 2006). There is limited information on males because they are entirely aquatic and do not return to nesting beaches, unlike females. However, in other species, like green turtles, males are often smaller than females (Godley et al. 2002). The largest leatherback turtle recorded was a dead stranded male on Harlech beach in Gwynedd, Wales, with a weight of 916 kg and a CCL of 256.6 cm (Eckert and Luginbuhl 1988). Thus, there is the possibility of females being even larger. On September 26, 2009, at Ko Phra Thong in Phang Nga province, Thailand, a largest leatherback turtle with a CCL of 220 cm and a curved carapace width (CCW) of 180 cm was observed. In contrast, on May 29, 2014, at Thai Mueang, also in Phang Nga province, a smaller individual was recorded with a CCL of 70 cm and a CCW of 116 cm.

The leatherback is the only sea turtle species that lacks scales and a hard shell, being covered by leathery skin instead (Spotila and Tomillo 2015). The color is black to bluish‐black with white‐pink speckling along the body (Robinson and Paladino 2013; Spotila and Tomillo 2015). There are five dorsal ridges on the carapace along the cranial to caudal position, with two additional ridges at the margins, while two to five ridges are present on the plastron (Spotila and Tomillo 2015). Unlike other sea turtles, no claws are present on the front or rear flippers (Paladino and Morreale 2001). The leatherback has the largest front flippers, often equal to or exceeding half the carapace length (James 2001; Paladino and Morreale 2001; Spotila and Tomillo 2015). The large flippers and the cranio‐caudal carapace ridges make them fast swimmers, which facilitates long‐distance migrations. The other distinctive feature of the leatherback turtle is the many backward‐pointing spines in the throat and esophagus, called keratinous papillae (Spotila and Tomillo 2015). These structures are adapted for a diet of soft‐bodied invertebrates such as jellyfish and other cnidarians to help retain the prey during the expulsion of seawater (Paladino and Morreale 2001).

### 2.2 Distribution of Leatherback Turtles

**FIGURE 1:**
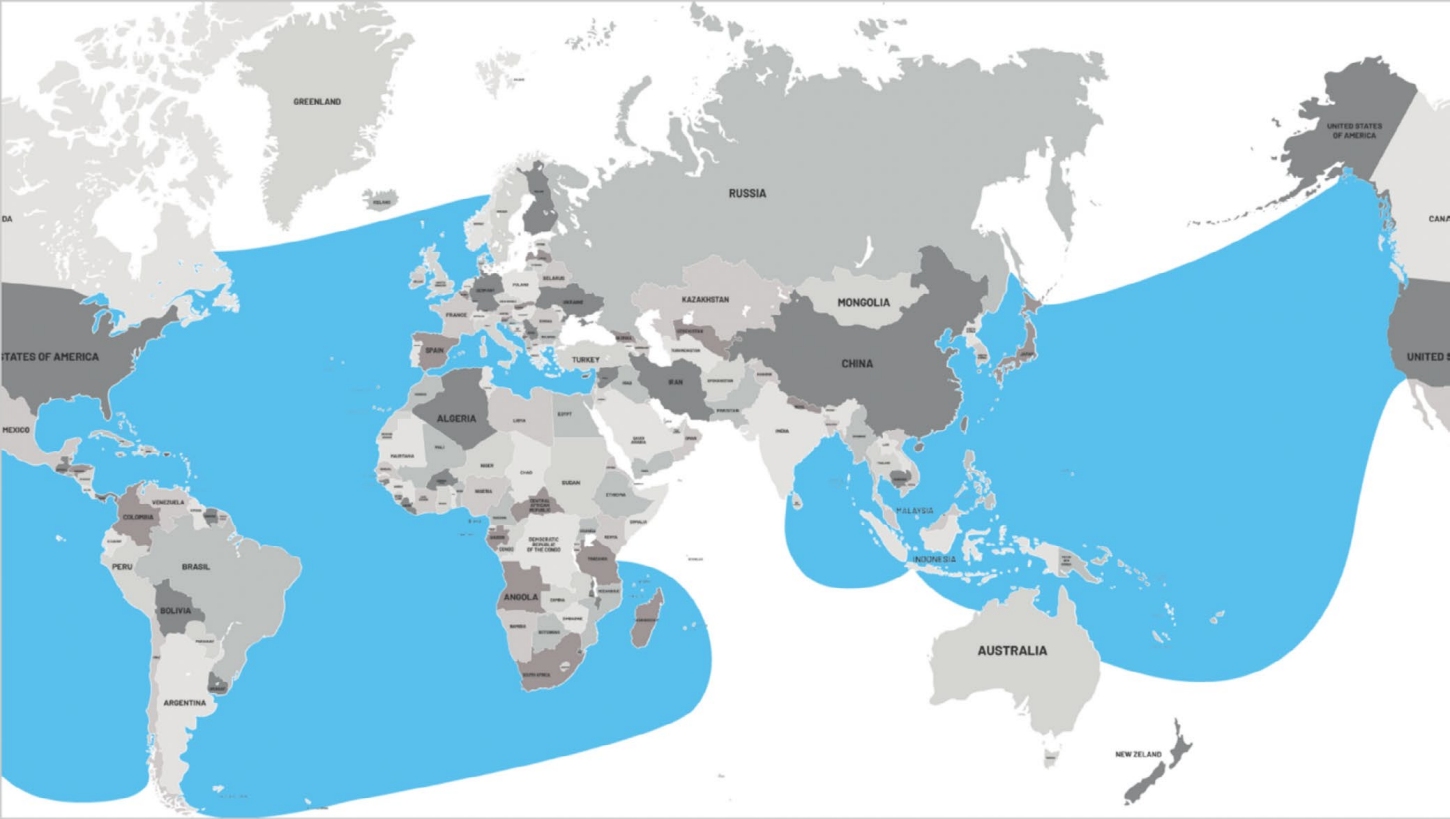
Global map of leatherback turtle distribution (blue areas). Figure reproduced using data from the International Union for the Conservation of Nature (IUCN, https://www.iucnredlist.org/species/6494/43526147).

The leatherback turtle has the largest distribution range (Wyneken et al. 2013), with seven subpopulations found in oceans spanning tropical and temperate waters of the Atlantic, Pacific, and Indian Oceans (Wallace et al. 2010) (Figure 1). In the Atlantic Ocean, leatherback turtles are found as far north as Newfoundland in Canada and the northeastern Atlantic near the British Isles. In the South Atlantic, they range from the southern coast of South America to the southwestern coast of Africa. In the Western Atlantic, leatherback turtles are present along the eastern coast of the United States, the Gulf of Mexico, the Caribbean Sea, and the northeastern coast of South America. In the Eastern Atlantic, they are found along the western coast of Africa from Mauritania to Angola. In the Pacific Ocean, they range from Japan across the North Pacific to the western coast of North America and up to Alaska. In the South Pacific, leatherback turtles are found along the southeastern coast of Australia to New Zealand and across the South Pacific to South America. In the Western Pacific, significant populations are found in areas such as Indonesia, Papua New Guinea, and the Philippines. In the Eastern Pacific, they are present along the coasts of Central and South America, from Mexico down to Chile. The leatherback turtle inhabits the western Indian Ocean, including the waters off the eastern coast of Africa, Madagascar, and the Seychelles. They are also found in the eastern Indian Ocean, including the waters around Sri Lanka, the Andaman and Nicobar Islands, and Western Australia. Populations of leatherback turtles in the Pacific Ocean have rapidly decreased over the past two decades, whereas those in the North Atlantic appear stable or increasing (Bailey et al. 2012). Related in part to high fisheries bycatch across the world's oceans (Hays et al. 2003; Lewison et al. 2004), the Atlantic and Pacific population trajectories show markedly different patterns (Saba et al. 2008; Spotila et al. 2000). In Thailand, stranded leatherback turtles have been found along both the Andaman Sea and the Gulf of Thailand. However, leatherback turtle nesting sites have only been discovered along the west coast of Phuket and Phang Nga. No reports of nesting sites have been found in other areas or in the Gulf of Thailand.

**FIGURE 2:**
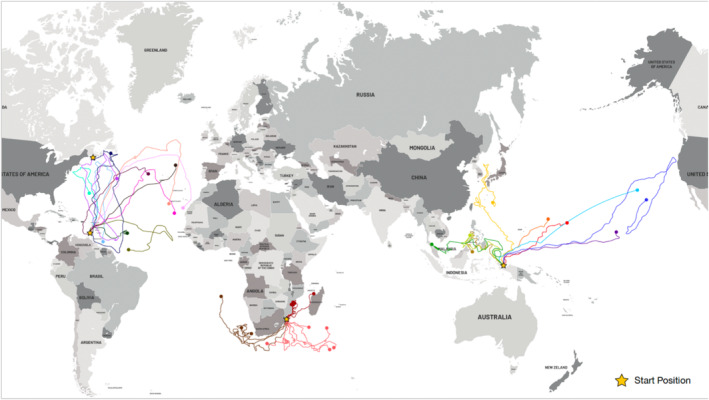
Migration routes of individual leatherback sea turtles. Figure reproduced from several sources (James et al. 2005a; 2005b; Hays et al. 2006; Robinson et al. 2016).

Evidence of leatherback turtle trans‐Pacific migration from the West Pacific Ocean, Japan, to the northern East Pacific, North America, has been demonstrated using satellite telemetry (Benson et al. 2007). Nine leatherback turtles were tracked for 111–695 days from one of the largest remaining western Pacific leatherback turtle nesting beaches (Jamursba—Medi, Papua, Indonesia) to the tropical waters of the Philippines and Malaysia, into the Sea of Japan, and across the equatorial Pacific to temperate waters of North America. In another study, Robinson et al. (2016) reported the movements of 16 post‐nesting leatherback turtles over 2 years from the iSimangaliso Wetland Park, South Africa, showing three migratory behaviors, with individuals traveling distances of up to 10,000 km toward the South Atlantic Ocean, the Western Indian Ocean, or the Mozambique Channel (Figure 2). There is a record of an individual migrating about 10,000 km from Matura beach (Trinidad) to Iskenderun Bay (Mediterranean) (Sönmez et al. 2008). Leatherback turtles can swim up to 100 km daily, contributing to their migratory ability (Hays et al. 2006). The habitat selection of leatherbacks shows a strong seasonal pattern. During the summer and early autumn, most leatherbacks remain in temperate latitudes, while in the late autumn, winter, and spring, they shift to subtropical and tropical habitats (Dodge et al. 2014).

### 2.3 Life Cycle and Behavior

Leatherback turtles grow rapidly during the early stages of life, about 32 cm per year based on measures of standard curved carapace length (Jones et al. 2011). Growth in adult nesting females slows to only 0.2 cm per year based on straight carapace length measures (Price et al. 2004). Leatherback turtles consume about 73% of their weight daily, feeding exclusively on soft‐bodied invertebrates like jellyfish and tunicates (Paladino and Morreale 2001). They mature relatively slowly, although estimates vary due to a lack of robust data (12–14 years (Dutton et al. 2005), 13–14 years (Girondot et al. 2021), 16.1 years (Jones et al. 2011), 24.5–29 years of age (Avens et al. 2009)) In one example, hatchling turtles returned to their birth site on Sandy Point Beach, St. Croix, to lay eggs as adults after around 12–14 years of age (Dutton et al. 2005). By contrast, leatherback turtles in captivity have been shown to reach sexual maturity as young as 5 years of age (Girondot et al. 2021; Wyneken et al. 2013). Longevity data are also limited, but it appears the leatherback turtle has the longest lifespan among sea turtle species. Based on gene promoter CpG methylation density, leatherback turtles are estimated to live up to 90 years, longer than other sea turtle species that live about 50–60 years (Mayne et al. 2020).

**FIGURE 3:**
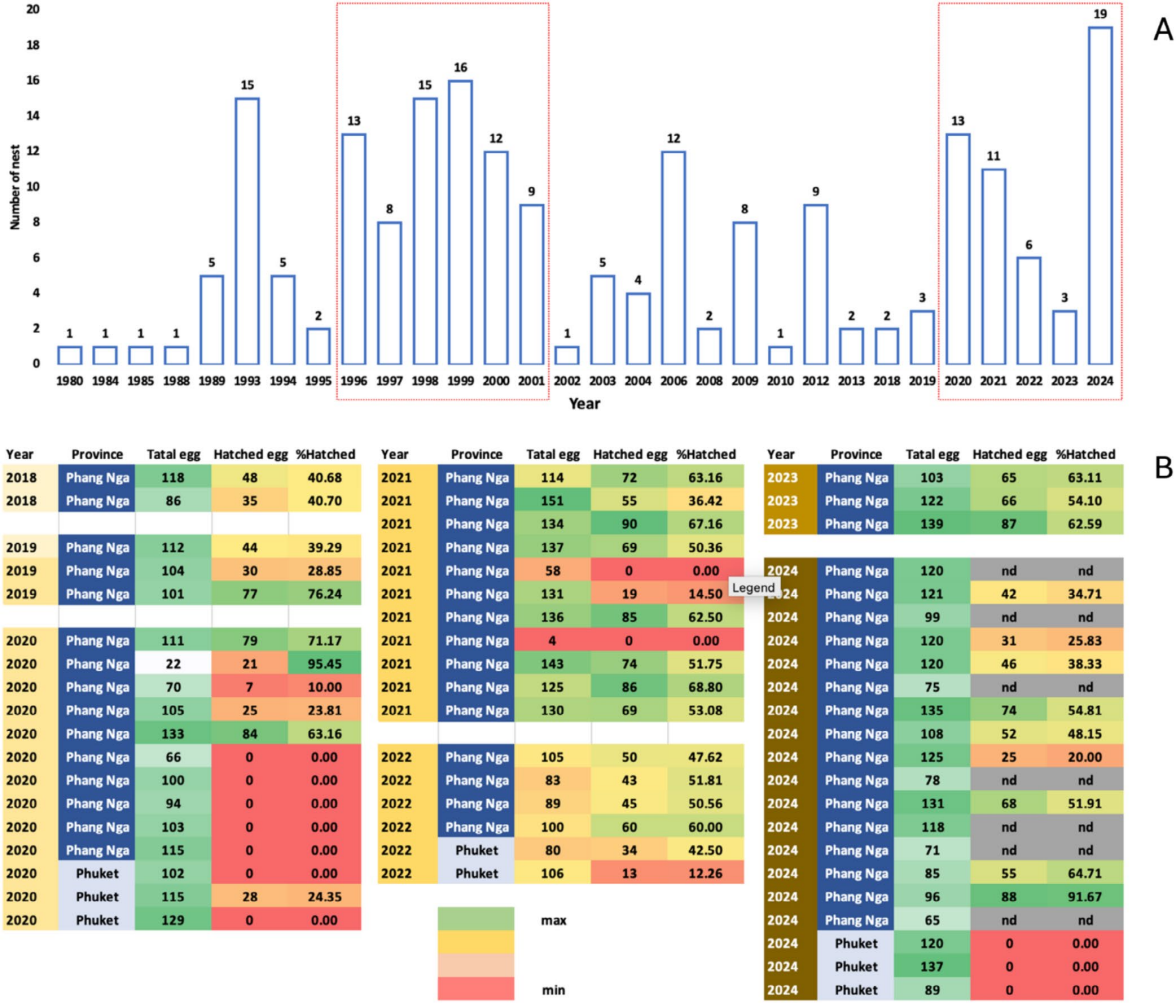
Number of leatherback turtle nests recorded in Thailand from 1980 to 2024 (A). Details of the number of eggs, hatched eggs, and the percentage of hatching turtles in Thailand between 2018 and 2024 (B). Red boxes indicate years that turtles consistently laid eggs, with a high number of nests. Data were sourced from (Wongfu et al. 2022, Resources Department of Marine and Coastal Resources, 2014) and Dr. Kongkiat Kittiwattanawong, and are owned by the Department of Marine and Coastal Resources, Thailand.

Like other sea turtles, adult female leatherbacks are seasonal nesters that exhibit natal homing behavior (Wyneken et al. 2013). Females return to their birthplace to lay several clusters of eggs, while male leatherbacks spend their entire lives offshore (Robinson and Paladino 2013; Wyneken et al. 2013). The gravid female usually lays 3–10 clusters of eggs for each nesting season, with 60–eggs per nest (Wallace et al. 2013). At Playa Grande, Costa Rica, the average clutch size and number of clutches a female laid per season from 2004 to 2007 were 62 ± 10 eggs and 9.5 ± 1.3 clutches, respectively (Santidrián Tomillo et al. 2009). The average time for females to return to a nesting site each season (interseasonal interval) can vary by individual and location depending on foraging success or food availability, oceanographic conditions, and environmental and human‐induced factors (Chua and Furtado 1988; Ferraroli et al. 2004; Hays et al. 2006). The interseasonal interval was about 2 years in Malaysian waters (Chua and Furtado 1988) or 2–3 years in Canada and the northeastern Atlantic (James et al. 2005b; Price et al. 2004). In Thailand, records between 1980 and 2024 from the Department of Marine and Coastal Resources (DMCR) indicate that female leatherback turtles return to their nesting sites at yearly intervals. For example, between 1996–2001 and 2020–2024, turtles consistently laid eggs every year, with a high number of nests (Figure 3A). However, some years had no nests, while others had only one or two (Resources Department of Marine and Coastal Resources 2014; Wongfu et al. 2022); records before 2018 were not as detailed. Presently, there are five main beaches in Thailand that serve as nesting sites for leatherback turtles. These include three beaches in Phang Nga—Khuk Khak Beach, Bor Dan Beach, and Bang Kwan Beach—and two beaches in Phuket—Sai Keaw Beach and Nai Thon Beach. As of 2010, global leatherback turtle nesting abundance was estimated at approximately 54,262 nests, representing a 40% decline from the 90,599 nests reported in the mid‐20th century (Wallace et al. 2013). Compared to other regions, the number of nests in Thailand is relatively low. For instance, in Sri Lanka, the nesting population is estimated at 100–200 nests per year, while in the Andaman and Nicobar Islands, approximately 400–600 nests are recorded annually (Hamann et al. 2006). A report in 2017 documented an average annual nesting count along the Pacific coast of Costa Rica over a five‐year period, with approximately 14 nests at Naranjo Beach, 14 nests at Cabuyal Beach, 10 nests at Nombre de Jesús, 23 nests at Ostional, and 4 nests at Caletas (Santidrián‐Tomillo et al. 2017). More recently, data from Grenada, West Indies, indicate an average of 81 nests per year between 2015 and 2019 (Charles et al. 2023).

Hendrickson and Balasingam (1966) identified five consistent factors that characterize nesting beaches: coarse‐grained sand, a steep and sloping littoral zone, an obstacle‐free approach, proximity to deep water, and an influence of oceanic currents on the coast. Strong waves and high tides assist females in arriving on the beach and reaching high, dry sand with less energy spent crawling (Reina et al. 2002). Key nesting sites for leatherback turtles are found in the Western Atlantic (Trinidad and Tobago), the southeastern United States (Florida), French Guiana; Eastern Atlantic (Gabon and other parts of West Africa); Western Atlantic (Brazil); Western Pacific (Indonesia, Solomon Islands, and Papua New Guinea), Eastern Pacific (Mexico and Costa Rica, and Indian Ocean), Andaman and Nicobar Islands, Sri Lanka, and a few beaches in Malaysia and South Africa (Santidrián‐Tomillo et al. 2017; Vargas et al. 2016; Wallace et al. 2010). In Thailand, over the past 44 years, nesting sites have been found near Phang Nga and Phuket along the Andaman Sea but not on other beaches.

The nesting season of leatherback turtles varies depending on geographical location (Kaewmong et al. 2022; Santidrián‐Tomillo et al. 2017; Wallace et al. 2010). While there is some overlap, different populations have distinct nesting seasons influenced by local environmental conditions. For example, nesting typically is observed from March to July in the Western Atlantic (Caribbean, Central America), November to April in the Eastern Atlantic (West Africa), November to February in the Western Pacific (Indonesia, Solomon Islands), October to February in the Eastern Pacific (Central America), and November to March in the Indian Ocean and Southeast Asia (Andaman and Nicobar Islands). Data from 1980 to 2020 in Thailand indicate that the nesting season starts in November and lasts until February; however, since 2020, the turtle nesting season has been extended from October to July or August (Wongfu et al. 2022).

After laying, eggs incubate for 55–60 days before hatching (Wyneken et al. 2013). Out of thousands of eggs laid, only 50% hatch (lower than other marine turtle species), and just 2% of those survive (Rafferty et al. 2011). The hatching success rate varies among locations and can be affected by factors such as precipitation and temperature (Choi et al. 2020; Rafferty et al. 2011). Data between 2018 and 2024 from the Department of Marine and Coastal Resources, Thailand (Wongfu et al. 2022) showed that the hatching rate ranged from 0% to 95.5% in 50 nests with data (out of 57 total) (Figure 3B). Twelve out of the 50 nests (25%) had no hatched turtles, while 17 nests (34%) had a hatching rate lower than 50%. Only two nests had a hatching rate over 80%, and 19 had a hatching rate between 50% and 80%. These data confirm that leatherback turtles in Thailand also have low hatching rates. In 2024, three nests out of 19 did not have any hatched turtles and were located on the same beach in Phuket, while 16 out of 19 nests in Phang Nga did hatch, possibly indicating a location effect (Rafferty et al. 2011). While seasonal effects cannot be managed by people, location effects can be, so better protection of nesting sites can aid sea turtle survival. In addition, many countries, including Thailand and Costa Rica, have protocols for leatherback turtle nest relocations (Furler 2005). Indeed, the researcher found no adverse effects on hatching rates between relocated leatherback turtle nests (*n* = 8) compared to nonrelocated nests (*n* = 8), and relocated nests had a lower rate of non‐developed eggs (Kaewmong et al. 2022). Therefore, relocating leatherback turtle eggs to more favorable locations could be an effective conservation strategy. This outcome is consistent with previous studies that recommend relocating sea turtle nests (Furler 2005; Reboul et al. 2021). It is known that all sea turtles have temperature‐dependent sex determination (TSD), with cooler temperatures producing males and higher temperatures yielding females (Ewert et al. 1994; Janzen 1994; Miller 2017), a factor that could be severely impacted by climate change and rising seawater temperatures. Globally, studies have indicated a female‐biased sex ratio in many leatherback populations. For instance, data in 2023 from 31 loggerhead rookeries, 14 green rookeries, nine leatherback rookeries, five hawksbill rookeries, three olive ridley rookeries, one Kemp's ridley rookery, and one flatback rookery estimated that in situ clutches were approximately 90% female, while hatchery clutches produced around 64% females (Laloë et al. 2024). These findings raise concerns about potential long‐term impacts on population dynamics, especially under ongoing climate change. Currently, there is a lack of data on the sex ratios of leatherback turtle hatchlings in Thailand. This gap is primarily due to the challenges associated with determining the sex of hatchlings, as they do not exhibit external sexual dimorphism traits. Consequently, direct sex ratio assessments in Thai populations are limited. However, anecdotal observations suggest a predominance of female hatchlings during certain seasons, but comprehensive data are lacking. Given the critical conservation status of leatherback turtles in Thailand, it is imperative to conduct detailed studies to assess hatchling sex ratios. Such data would provide valuable insights into the population structure and inform targeted conservation strategies.

### 2.4 Foraging Sites

Post‐nesting, leatherback females typically forage in pelagic (open ocean) zones, often far from coastal areas (Dodge et al. 2014), and return to feeding grounds in more northern, temperate waters to prepare for the next nesting season in tropical seas (Ferraroli et al. 2004; Hays et al. 2004a; 2004b; James 2004; Luschi et al. 2003). These zones are rich in jellyfish, their primary prey, which are abundant in the mid‐to‐surface layers of the ocean (Sato 2017, Witt et al. 2007). While leatherbacks are often seen near the surface, they are capable of deep dives, sometimes exceeding 1000–1200 m (Eckert et al. 1989; Hays et al. 2004b). This allows them to exploit prey found at various depths, depending on the availability and distribution of food. Leatherback turtles exhibit greater path sinuosity as water depth decreases in both temperate and tropical shelf habitats. This pattern aligns with the observed rise in gelatinous zooplankton biomass in shallower waters (Tanabe et al. 2023). Leatherbacks are unique among sea turtles for their ability to maintain a body temperature higher than that of the surrounding water (Jonsen et al. 2007). This allows them to forage in colder waters, including those of temperate and subpolar regions in the North Atlantic, North Pacific, and Southern Oceans (Dodge et al. 2014; Witt et al. 2007). Leatherbacks are known to migrate to high‐latitude regions during the summer months to exploit seasonal blooms of jellyfish. Bailey and colleagues (Bailey et al. 2012) demonstrated distinctly different patterns between leatherback turtles in the North Atlantic and those in the Eastern Pacific, both of which feed on gelatinous zooplankton that are infrequently found in high densities. In the Atlantic, travel speeds exhibited two distinct modes, suggesting successful foraging at lower speeds (< 15 km/day) and transit at higher speeds (20–45 km/day). This extensive range highlights their adaptability and the critical importance of international conservation efforts to protect migratory routes and nesting sites. However, currently, there are no specific data identifying designated foraging areas for leatherback turtles within Thailand's territorial waters. Leatherbacks are known for their extensive migratory behavior and do not exhibit site fidelity to specific foraging grounds, unlike some other marine species. In Thailand, existing reports primarily document nesting activities along the Andaman Sea coast, particularly in Phuket and Phang Nga provinces. Additionally, there have been instances of leatherback turtle stranded records along the Thai coast both in the Andaman Sea and the Gulf of Thailand (Table 1). Given the absence of localized foraging data, it would be beneficial for future research to focus on identifying potential feeding habitats within Thailand and its adjacent regions. Implementing satellite telemetry studies could provide insights into the migratory routes and foraging behaviors of leatherbacks frequenting Thai waters. Such information would be invaluable for developing targeted conservation strategies and mitigating threats from fisheries interactions.

**TABLE 1:** The information of 44 stranded leatherback turtles in Thailand from 1993 to 2024.

Date	Province	Sea	Sex	CCl (cm)	CCW (cm)
4‐Jul‐1993	Rayong	Gulf of Thailand	M	190	nd
28‐Jul‐1994	Trat	Gulf of Thailand	U	nd	nd
7‐Jan‐1995	Phang Nga	Andaman Sea	U	114	160
29‐Dec‐1996	Phang Nga	Andaman Sea	U	169	123
5‐Oct‐1998	Phang Nga	Andaman Sea	U	nd	nd
16‐Mar‐1999	Phang Nga	Andaman Sea	U	nd	nd
16‐Mar‐1999	Phang Nga	Andaman Sea	U	nd	nd
16‐Mar‐1999	Phang Nga	Andaman Sea	U	nd	nd
16‐Mar‐1999	Phang Nga	Andaman Sea	U	nd	nd
16‐Mar‐1999	Phang Nga	Andaman Sea	U	nd	nd
7‐Feb‐2000	Phang Nga	Andaman Sea	U	nd	nd
9‐Jun‐2002	Phang Nga	Andaman Sea	U	nd	nd
23‐Sep‐2003	Chumphon	Gulf of Thailand	U	200	100
15‐Oct‐2007	Phang Nga	Andaman Sea	U	200	200
29‐Jun‐2008	Phuket	Andaman Sea	U	180	115
4‐Jul‐2008	Chonburi	Gulf of Thailand	F	160	79
27‐Nov‐2008	Prachuap Khiri Khan	Gulf of Thailand	F	155	110
30‐Jan‐2009	Phuket	Andaman Sea	F	131	171
26‐Sep‐2009	Nakhon Si Thammarat	Gulf of Thailand	U	220	167
30‐Sep‐2010	Phang Nga	Andaman Sea	M	70	116
2‐Mar‐2011	Phuket	Andaman Sea	U	200	150
28‐Feb‐2012	Rayong	Gulf of Thailand	U	120	100
29‐May‐2014	Phuket	Andaman Sea	U	200	115
30‐Jun‐2014	Phang Nga	Andaman Sea	U	200	170
22‐Jul‐2014	Satun	Andaman Sea	F	96	120
23‐Feb‐2015	Phang Nga	Andaman Sea	U	nd	nd
28‐Jul‐2015	Samut Songkhram	Gulf of Thailand	M	96	140
6‐Aug‐2015	Ranong	Andaman Sea	U	nd	nd
21‐Feb‐2016	Phang Nga	Andaman Sea	U	91	148
26‐Dec‐2016	Nakhon Si Thammarat	Gulf of Thailand	F	125	105
28‐Jun‐2017	Chonburi	Gulf of Thailand	F	170	110
19‐Mar‐2018	Chumphon	Gulf of Thailand	F	nd	nd
26‐Sep‐2018	Rayong	Gulf of Thailand	U	137	97
19‐Mar‐2019	Ranong	Gulf of Thailand	F	208	185
12‐Jul‐2019	Rayong	Gulf of Thailand	F	123	79
4‐Jan‐2020	Phang Nga	Andaman Sea	F	80	100
30‐Jan‐2022	Krabi	Andaman Sea	U	nd	nd
15‐Feb‐2022	Chumphon	Gulf of Thailand	U	82	145
29‐Jun‐2022	Rayong	Gulf of Thailand	U	nd	nd
7‐Dec‐2023	Trang	Andaman Sea	U	nd	nd
13‐Dec‐2023	Phang Nga	Andaman Sea	F	220	180
4‐Jan‐2024	Chumphon	Gulf of Thailand	F	115	80
22‐Jan‐2024	Phang Nga	Andaman Sea	F	140	105
8‐Feb‐2024	Bangkok	Gulf of Thailand	M	102	146

*Note:* The data were provided by Dr. Patcharaporn Kaewmong and Dr. Kongkiat Kittiwattanawong, the authorized person from the Department of Marine and Coastal Resources, Thailand.

Abbreviations: CCL, curved carapace length; CCW, over carapace width; F, female; M, male; nd, no data; U, unknown.

## 3 Genetic Diversity of the Leatherback Sea Turtle

Genetic monitoring is vital for sea turtle conservation, offering insights into genetic diversity, population structure, and migration patterns. These data help track biological changes, identify distinct populations, and address human activities and climate change threats. Currently, genetic information is available for all seven sea turtle species: leatherback (Dutton et al. 1999; Dutton et al. 2007; Dutton et al. 2013; Vargas et al. 2008; Wongfu et al. 2022), green (Bass et al. 1996; Jordão et al. 2015), loggerhead (Baltazar‐Soares et al. 2020), hawksbill (Bass et al. 1996; González‐Garza et al. 2015; Vargas et al. 2016), olive ridley (Aggarwal et al. 2008; Madduppa et al. 2021), kemp's ridley (Camacho‐Sánchez et al. 2022; Lamont et al. 2021), and flatback (FitzSimmons et al. 2020). Genetic monitoring thus is crucial for developing and prioritizing effective conservation strategies to ensure the survival of these endangered species where mitigating policies are most needed.

In population genetic studies of leatherback turtles, the displacement loop (D‐loop) mitochondrial DNA, or control region, is one of the most commonly used markers (Dutton et al. 1999; Dutton et al. 2013; Vargas et al. 2008; Yoshikawa et al. 2016). This maternally inherited non‐coding region has been used to trace maternal lineages, evaluate genetic diversity and population structure, and detect patterns of gene flow among nesting populations of sea turtles. These are observed through nucleotide variation within the consensus sequences, called haplotypes. Variation among global haplotypes of leatherback turtles was first investigated by Dutton et al. (1999). Only 11 mtDNA control region haplotypes with nine variable sites were found for 10 nesting locations worldwide, including five nesting sites in the Atlantic region (Florida, Costa Rica, Trinidad, Guianas, and U.S. Virgin Islands), three nesting sites from the Indo‐Pacific region (South Africa, Malaysia, and Solomon Islands) and two nesting sites from the East Pacific region (Mexico and Costa Rica). In that study, mtDNA primers LTCM1 and HDCM1 were used to amplify 496 bp of control region sequences. The nesting populations were found to be genetically subdivided to a high degree (global FST = 0.415), reflecting strong natal homing behavior. The combined haplotype diversity from all nesting sites was 0.67, considered low compared to other sea turtle populations studied in the same period. For example, global nesting sites of olive ridley turtles had a haplotype diversity of 0.81 using similar lengths of mtDNA control region sequences at 470 bp (Bowen et al. 1997). Green turtle populations in the Atlantic Ocean and Mediterranean Sea also had higher haplotype diversity at 0.83 using the length of mtDNA at 487 bp (Bass et al. 1996).

In addition to low global mtDNA haplotype diversity, leatherbacks also have shallow topography in phylogenetic analysis and no clear separation of matriarchal lineage across ocean basins (Bowen and Karl 2007; Dutton et al. 1999). This suggests a recent radiation of the species hypothesized to occur from the Indo‐Pacific region through haplotype D, which is considered a haplotype in the middle of the network. The sharing of haplotype A also occurred among subpopulations in the Indo‐Pacific and Atlantic Oceans. The highest haplotype diversity was observed in western Pacific Malaysian nesting sites (Nh = 4, *h* = 0.806 ± 0.089) consisting of haplotypes A, D, E, and H (Dutton et al. 1999). Unfortunately, this area is also experiencing one of the most dramatic population decreases. Those haplotypes were also found in the Solomon Islands and Papua New Guinea. For areas around the Solomon Islands, haplotype I was found to be specific only to nesting populations (Dutton et al. 1999; Dutton et al. 2007). These areas are close to the Thai Andaman Sea, known as a nesting site for leatherback turtles, although, at that time, the population was not included in any study.

In recent studies, LCM15328 and H950g primers are increasingly used in sea turtle genetic research, including the leatherback, as they can amplify longer mtDNA control regions at 711 bp (Allard et al. 1994; Vargas et al. 2008), thus providing better mtDNA haplotype resolution. Previously, Vargas et al. (2008) conducted a genetic diversity study of leatherbacks along the Brazilian coast. Using 496 bp sequences, only two haplotypes (A and C) were found for the nesting population of Espírito Santo State. More variable sites were found for haplotype A using a longer sequence at 711 bp, splitting into A1, A2, A3, and A4. In that study, for bycatch and stranding individuals (foraging group), seven haplotypes were found, including haplotypes A1, A2, A3, A4, C, D, and I. Foraging groups usually consist of individuals originating from different nesting locations, resulting in the higher genetic diversity observed in feeding grounds (Bowen and Karl 2007). Interestingly, the finding of haplotype I originating from the Solomon Islands (Pacific Ocean) in the Brazilian feeding grounds could indicate a hidden nesting population within the Atlantic Ocean, and perhaps some individuals are migrating long distances from western Pacific nesting sites to the Atlantic Ocean foraging grounds. In addition, there have been occurrences of stranded leatherbacks having haplotype I around Japan, from Hokkaido to Okinawa, which is not known as a nesting site for this species (Yoshikawa et al. 2016). Thus, it appears there is more variability in the maternal lineages in the feeding grounds compared to the nesting sites.

**FIGURE 4:**
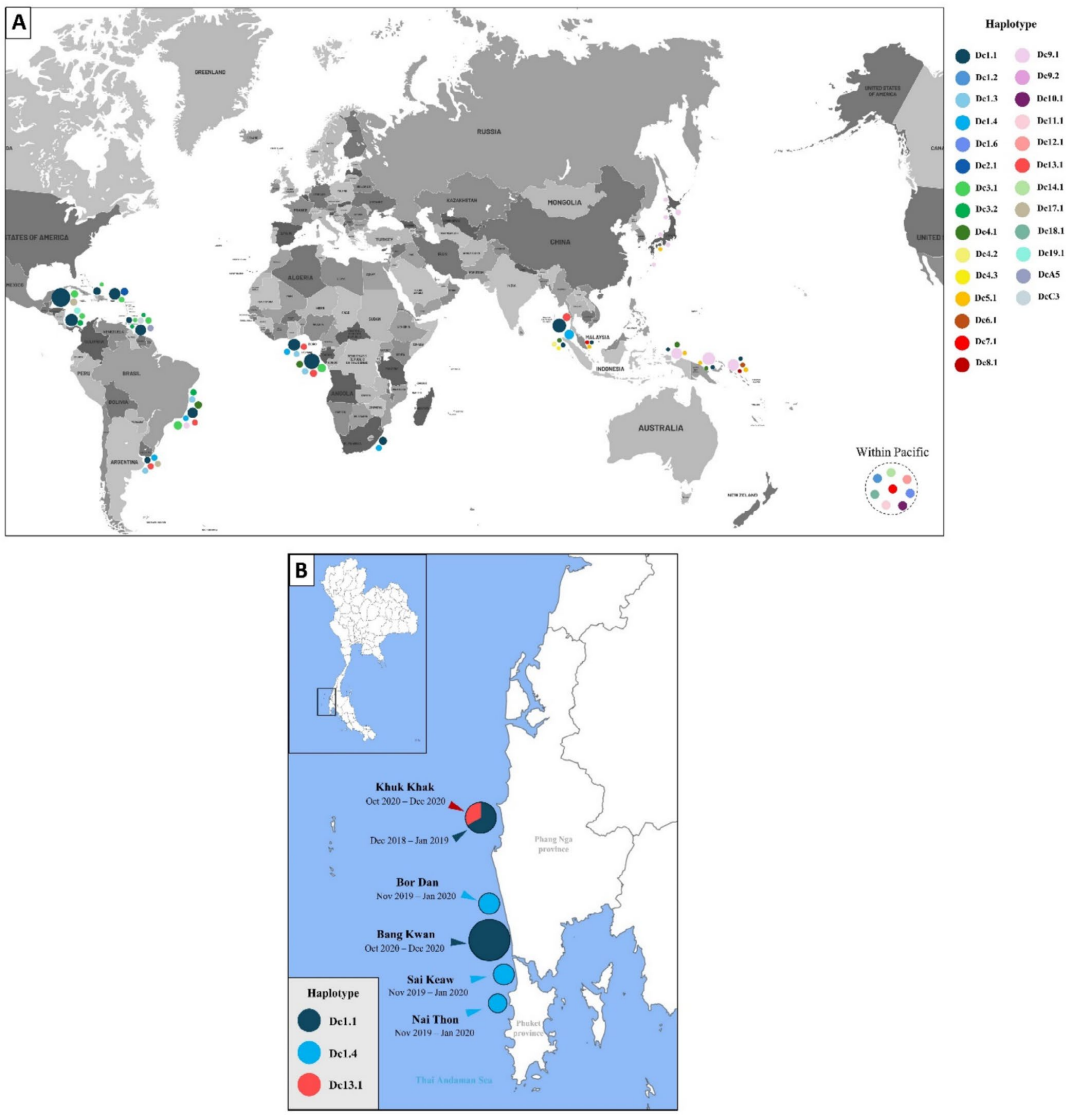
Distribution of 27 global haplotypes of the leatherback sea turtle (A) (Dutton et al. 2013; Vélez‐Rubio et al. 2023; Wongfu et al. 2022; Yoshikawa et al. 2016). Three mtDNA haplotypes (Dc1.1, Dc1.4 and Dc13.1), similar to Dutton et al. (2013), in nesting populations of leatherback turtles in the Thai Andaman Sea from 2018 to 2020 (B). Figure reproduced from Wongfu et al. (2022).

The recent study in Thailand analyzed a total of 1428 sequences of mtDNA control region from worldwide populations of leatherback turtles collected from nesting sites and pelagic aggregating devices deposited into the NCBI database (Wongfu et al. 2022). Based on 658 bp mtDNA control region sequences, the combined haplotype diversity was low, 0.43 compared to 0.67 reported in an earlier study Dutton et al. (1999). The total number of haplotypes also was higher, up to 27 haplotypes compared to earlier findings (Figure 4A) (Dutton et al. 1999; Dutton et al. 2007; Dutton et al. 2013; Vargas et al. 2008). This is still considered a low number of haplotypes; however, it is noted when compared to other sea turtle species (Wongfu et al. 2022). For example, 88 haplotypes from 1983 hawksbill turtles were found in global rookeries (Arantes et al. 2020). In Thai Andaman Sea rookeries, three haplotypes were found from five nesting beaches (Figure 4B) (Wongfu et al. 2022), (haplotype 1, 2 and 3) that resembled haplotype Dc1.4 (haplotype A4), Dc1.1 (haplotype A1) and Dc13.1 (new haplotype) described previously by Dutton et al. (2013). These haplotypes are not restricted to the Thai Andaman Sea but occur in other areas as well. Haplotype Dc1.1, while recognized as a common haplotype for the Atlantic Ocean, is also found in the Pacific Ocean but only in a small number, just 1–3 individuals found in Malaysia and the Solomon Islands (Dutton et al. 2007). Haplotype Dc1.4 has been found in Ghana rookeries in the Atlantic Ocean, South African rookeries in the Indian Ocean (Dutton et al. 2013), and in a pelagic individual in Brazilian waters (Vargas et al. 2008).

Haplotype Dc13.1 was a new haplotype found in West African rookeries (Dutton et al. 2013). Other than that area and Thai Andaman Sea rookeries, this haplotype has not been found elsewhere. In Thai Andaman Sea rookeries, this haplotype (Dc13.1) was only found in one nest on Khuk Khak beach in the year 2020 from a total of nine nests (Figure 4B) (Wongfu et al. 2022). The other eight nests on Bang Kwan Beach that year exhibited haplotype Dc1.1; after that year, it was never detected again at those sites. This may be because a lack of nest‐site‐specific behaviors has been observed for some leatherback females (Bowen and Karl 2007), and this nesting female with haplotype Dc13.1 likely originated from other areas. Thus, there may be more haplotypes in hidden nesting sites and foraging grounds that have not been investigated around the Pacific region, including Thailand's coasts. The haplotypes expected to be found around these areas include haplotype Dc5.1 (haplotype E) and Dc8.1 (haplotype H) found in Malaysia (Dutton et al. 1999; Dutton et al. 2007) and haplotype Dc9.1 (haplotype I) from the Solomon Islands and Japan (Dutton et al. 2007; Yoshikawa et al. 2016).

Two mtDNA primers, LCM15328 and H950, are also used for leatherback population genetic studies along with microsatellite markers to provide a more comprehensive understanding of population dynamics, population structure, and paternity in leatherback turtles (Crim et al. 2002; Dutton et al. 2013; Roden et al. 2017; Vargas et al. 2022). We used eight loci of microsatellite markers to detect the population structure of turtles in nesting sites within Thai Andaman Sea rookeries (Wongfu et al. 2022). Five nesting beaches are divided into two genetic clusters (*K* = 2) in which the nesting populations from Bang Kwan Beach and some parts of Khuk Khak Beach showed the same genetic component. Interestingly, the PCA also showed the separation among these nesting beaches. The nesting population from Bang Kwan in 2020 and Khuk Khak beach in 2018 grouped together and appeared to be from the same parents as both also had the same mtDNA haplotype Dc1.1 (Wongfu et al. 2022). By contrast, the nesting population from Khuk Khak Beach in 2020 was from different parents with a different haplotype (Dc13.1) (Wongfu et al. 2022). That study included samples over 3 years only (2018 to 2020), so long‐term monitoring is still needed to determine population dynamics and observe genetic diversity changes over time.

## 4 Threats of the Leatherback Turtle

Leatherback turtles face numerous threats worldwide, significantly impacting their survival.

### 4.1 Habitat Loss and Degradation

Increasing construction of resorts, homes, and other infrastructure on nesting beaches is destroying critical nesting habitats of sea turtles, including leatherbacks (Maison 2006; Santidrián Tomillo et al. 2007; Sarti et al. 1996). Roads, seawalls, and other infrastructure can prevent female turtles from accessing suitable nesting sites or returning to the ocean after laying eggs. Beach erosion from both natural processes and human activities can wear away beaches, making them unsuitable for nesting. Light pollution from artificial sources disorients hatchlings, leading them away from the sea and increasing mortality rates (Lorne and Salmon 2007; Thums et al. 2016).

Habitat loss and degradation in Phuket and Phang Nga, Thailand, have significantly impacted leatherback turtles, particularly their nesting and reproductive success. Historically vital nesting grounds for leatherbacks, these areas have faced considerable challenges due to a combination of natural and human‐induced factors. Rapid urbanization and tourism expansion have led to extensive construction along beaches in Phuket and Phang Nga (Somphong et al. 2022). The development of resorts, restaurants, and other infrastructure has encroached on critical nesting habitats, leaving limited space for leatherbacks to lay their eggs. Notably, beachfront locations have been shown to raise hotel room rates by 13%–41% (Somphong et al. 2022). Additional sandy beach variables, such as beach length, width, and slope, also contribute to higher hotel prices (Somphong et al. 2022), emphasizing the competition for high‐quality sandy beaches between tourism and turtle nesting needs. Artificial lighting from coastal establishments further exacerbates the issue by disorienting nesting females and hatchlings, which significantly reduces reproductive success.

Human‐induced changes to the coastline, including the construction of hotels, resorts, seawalls, and jetties, have intensified natural erosion processes. A study examining 33 sandy beaches in Phuket revealed that eight locations experienced mild erosion between 2013 and 2021, with shoreline changes ranging from −4.10 to 5.47 m/year (Nidhinarangkoon et al. 2023). This research highlights how coastal urbanization and the development of structures along the shore influence beach morphology, further degrading critical nesting areas. Plastic pollution and debris on beaches pose additional threats to nesting females and hatchlings. High levels of human activity and waste accumulation discourage leatherbacks from nesting and degrade the quality of sand, which is vital for creating a suitable microenvironment for egg development. Climate change exacerbates these challenges. Rising sea levels and increased storm intensity threaten nesting habitats in Phuket and Phang Nga by causing frequent flooding and sand compaction. These effects can destroy nests or hinder successful egg incubation. Furthermore, changes to the beach profile due to erosion and flooding diminish the availability of sandy areas with the appropriate substrate and temperature for leatherback egg incubation. The combined impacts of urbanization, coastal modifications, pollution, and climate change have significantly degraded the nesting habitats of leatherback sea turtles in Phuket and Phang Nga. Efforts to mitigate these challenges should prioritize balancing tourism development with conservation initiatives, protecting sandy beach habitats, and addressing pollution and climate change effects. Such measures are essential for safeguarding the remaining nesting sites and supporting the recovery of leatherback populations in the region.

### 4.2 Water Pollution

Ocean water pollution is known to affect the survival of leatherbacks and other turtle species, while plastics in the marine environment are a growing environmental issue (Doyle 2007; Wilcox et al. 2018; Tanabe et al. 2023). Leatherback turtles primarily feed on jellyfish and often mistake plastic bags and other debris for food. Ingesting plastic can block the digestive system, leading to malnutrition, starvation, or death. In 2018, two datasets were analyzed, one comprised of necropsies on 246 sea turtles and another containing 706 records extracted from a national strandings database (Wilcox et al. 2018). Animals that died from known causes unrelated to plastic ingestion had less plastic in their guts compared to those that died from indeterminate causes or directly due to plastic ingestion (e.g., through gut impaction and perforation). The findings indicated a 50% probability of mortality once an animal had 14 pieces of plastic in its gut. Oil spills, heavy metals, and other toxic substances can also contaminate feeding grounds and accumulate in the body, leading to health issues, reduced reproductive success, and increased mortality (Esposito et al. 2023).

The nesting areas of leatherback turtles in Thailand, particularly in Phuket and Phang Nga, face significant environmental challenges due to tourism activities. Tourism is a cornerstone of the economy and reputation of these provinces, but it also contributes heavily to plastic debris along the coastline. This plastic pollution, especially microplastic debris, leads to marine environmental degradation and poses a threat to leatherback turtle habitats. The abundance of microplastics in marine environments is influenced by various factors, including wind currents, coastline geology, and human activities. A preliminary study conducted in 2019 on beach sediment samples from Kalim, Tri Trang, and Patong Beaches in Phuket (Akkajit et al. 2019) revealed that microplastic debris varied in abundance from 1 to 35 items per square meter, depending on the sampling location. Additionally, the average abundance of microplastics in sediment from six beaches in Phuket was found to be 188.3 ± 34.5 items per kilogram (Akkajit et al. 2021).

Further research focused on Mai Khao Beach, a critical nesting site for leatherback turtles, reported microplastic abundance in 2022 (Akkajit and Khongsang 2022). This study indicated that anthropogenic activities are likely sources of the microplastics, with mean abundances ranging from 44.08 to 68.7 items per kilogram for particles greater than 300 μm, and from 90.6 to 106.1 items per kilogram for particles in the 20–300 μm range. The highest microplastic levels were observed at Chalong Bay and Rawai Beach, which are popular tourist destinations and serve as departure points for tourists. Notably, the abundance of microplastics in the study area was significantly higher than at other sites investigated along the west coast of Phuket, underscoring the impact of tourism and associated human activities on the region's environmental health.

### 4.3 Fisheries Bycatch

Leatherback turtles are frequently caught in fishing gear such as longlines, gillnets, and trawls. This incidental capture, known as bycatch, can lead to injury or death. Pelagic longline fishing, a type of gear used in all the world's oceans, is directly associated with high rates of bycatch and varying mortality rates among sea turtles (Swimmer et al. 2017; Wallace et al. 2010; 2013). The mortality of sea turtles in fisheries bycatch was studied along the coast of Togo, West Africa, from 2012 to 2015 (Segniagbeto et al. 2017). This study reported that both site and net type significantly affected the number of dead turtles in fishing nets, but that a variance components estimation indicated that the site had a much higher effect than the net type.

**FIGURE 5:**
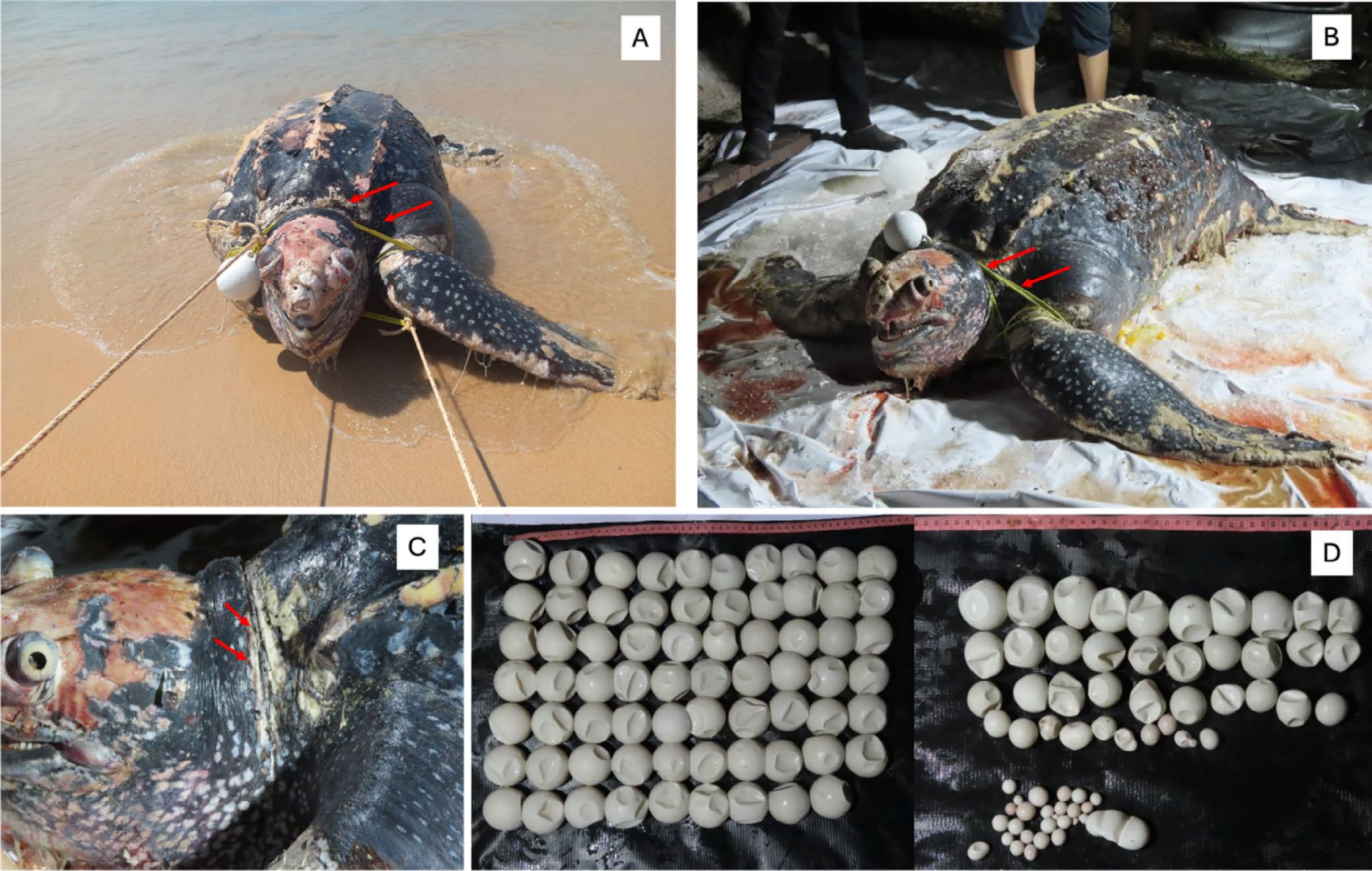
A stranded female leatherback turtle was found at Thai Mueang Beach, Phang Nga (A). A rope was tied around its neck and both front flippers (B, C). The autopsy did not reveal significant lesions due to decomposition of the carcass, but over a hundred eggs were found (D). However, the veterinarian believes that the cause of death was from a rope tied around its neck and front flippers (Photo by Patcharaporn Kaewmong).

“Fishing activities have significantly impacted many protected marine animals in Thailand. A survey conducted in 2016–2017 reported annual catches of marine megafauna, with respondents indicating a total of 419,821 rays, 28,920 sharks, 269 sea turtles, 47 small cetaceans, and 6 dugongs. Of these, 231,992 rays, 20,084 sharks, 246 sea turtles, 44 small cetaceans, and 5 dugongs were caught in the Gulf of Thailand, while 187,642 rays, 8836 sharks, 23 sea turtles, 3 small cetaceans, and 1 dugong were caught in the Andaman Sea (Svarachorn et al. 2023). The study also revealed that gillnets had the highest catch per unit effort for all megafauna groups in both sea areas, except for sea turtles, where pound nets had the highest catch per unit effort in the Gulf of Thailand. Among gillnets, crab gillnets showed the highest catch per unit effort for all groups except dugongs, for which ray gillnets were most impactful. When accounting for fishing effort, crab gillnets and shrimp trammel nets were responsible for the majority of the megafauna catch, with crab gillnets contributing 72%–95% of the annual estimated marine megafauna catch. Additionally, 46% of the interviewed fishers and 27% of all small‐scale fishers operating in Thai waters used crab gillnets, while shrimp trammel nets were used by 40% of interviewed fishers and 15% of small‐scale fishers (Svarachorn et al. 2023). To prevent the extirpation of threatened megafauna species that play vital roles in ecosystem resilience and productivity, restrictions on gillnet fishing efforts, particularly crab gillnets, are urgently needed. In early 2025 (January 22), a large female leatherback turtle with a CCL of 140 cm and a CCW of 105 cm was found stranded at Thai Mueang Beach, Phang Nga (Figure 5). A rope was tied around its neck and both front flippers, and the carcass was too decomposed to determine the exact cause of death. However, veterinarians believe the turtle likely died due to the rope, which was identified as part of a crab net. Tragically, this leatherback turtle was likely coming to lay eggs in the area. The autopsy revealed a significant number of eggs still in the body, indicating the start of the leatherback turtle nesting season. If the turtle had just begun laying eggs, it is possible that hundreds of eggs were lost. Each female leatherback turtle is capable of laying hundreds of eggs per nesting season.”

### 4.4 Climate Change

Climate change was reported to be an emerging threat to marine turtles 40 years ago, and although it initially received little attention, since 2007, research efforts have been continuously increasing (Boero et al. 2016). Factors associated with climate change affect marine turtles in both marine and coastal environments. Habitats are threatened as rising sea levels lead to the loss of nesting beaches. Rising global temperatures affect sand temperatures at nesting sites, influencing the sex ratio of hatchlings, with warmer sands tending to produce more females, potentially disrupting future breeding populations (Boero et al. 2016). Moreover, warmer sand influences hatchling conditions and provokes juveniles that are smaller in size to swim faster (Booth et al. 2004; Glen and Mrosovsky 2004; Simantiris 2024; Yao et al. 2022). There is no direct evidence that temperature rises threaten tropical jellyfish prey species, as it does with coral species (Purcell 2005), but this might be due to the current lack of observations (Boero et al. 2016). Other ways global warming might affect sea turtles are by altering migratory routes due to changes in environmental conditions and ocean currents, as well as altering the distribution of foraging areas.

The impact of climate change on leatherback turtles in Thailand remains uncertain due to a lack of comprehensive scientific studies. Additionally, the number of leatherback turtles nesting in Thailand is relatively low. However, some behaviors that have changed might be affected by climate change. Along the Andaman Sea coastline in southern Thailand, peak rainfall typically occurs between mid‐September and mid‐October. From November to March, the weather is generally considered optimal, with cooling winds tempering high summer temperatures, resulting in a more comfortable daily average of 26°C–32°C. Historically, leatherback turtles have been observed nesting starting in November. However, by late 2024, the Andaman coast experienced ongoing storms and heavy rainfall, likely linked to the effects of El Niño (Chueasa et al. 2024). As a result, no nesting activity was recorded during this period. The first nesting was observed only in mid‐January 2025.

## 5 Conservation Implications

Leatherback turtles face numerous threats that necessitate comprehensive conservation efforts. Habitat degradation, climate change, fisheries bycatch, and pollution significantly impact their populations. In Thailand, nesting sites along the Andaman coast are critical for this species, yet increasing coastal development and human activities threaten their reproductive success. Conservation strategies must prioritize habitat protection, including enforcing regulations to limit coastal infrastructure expansion and controlling artificial lighting, which disrupts nesting females and hatchling orientation. Moreover, fisheries bycatch remains a major concern, with high mortality rates associated with gillnets and trawl fisheries. Implementing bycatch reduction technologies can mitigate accidental captures. Additionally, engaging local fishing communities in sustainable practices and promoting awareness of leatherback conservation can enhance compliance with protective measures. Today, climate change poses a growing challenge, particularly with rising temperatures affecting hatchling sex ratios due to temperature‐dependent sex determination. Conservation efforts should include nest relocation to shaded areas or artificial cooling techniques to maintain balanced sex ratios. Furthermore, rising sea levels and increasing storm intensity threaten nesting beaches, underscoring the need for coastal habitat restoration and erosion control initiatives. In 2023, a pioneering conservation effort for leatherback turtles in Thailand was initiated (Kanghae et al. 2024). In this program, newly hatched leatherback sea turtles were randomly selected from two nests and successfully reared before their release. The study suggests that improvements in diet, water supply, and pond systems could enhance the outcomes of such head‐starting initiatives. This research provides valuable insights into local conservation strategies for leatherback turtles in Thailand.

For the genetic studies have highlighted the importance of maintaining global population connectivity to preserve genetic diversity. Conservation strategies should integrate genetic monitoring to identify critical nesting and foraging grounds, enabling targeted protection efforts. International collaboration is essential to address the transboundary nature of leatherback migrations, ensuring coordinated conservation efforts across regions. Presently, many international working groups for sea turtle conservation have been working hard to protect the sea turtle. For this, the conservation of leatherback turtles in Thailand and globally requires a multifaceted approach, combining habitat protection, fisheries management, climate adaptation, and genetic monitoring. Strengthening legal frameworks, enhancing community engagement, and fostering international partnerships are crucial to ensuring the long‐term survival of this species.

## 6 Conclusion

Knowledge from population genetics studies of leatherback turtles can provide critical insights into their biology and inform strategies to enhance conservation efforts. High genetic diversity is crucial for the adaptability and long‐term survival of species; presently, leatherback turtles have low genetic diversity (*h* = 0.43). Protecting diversity, ensuring connectivity between populations, and addressing region‐specific threats are essential components of effective conservation programs. Methods related to biological, behavioral, and genetic research are valuable tools in the global effort to conserve these majestic and highly migratory creatures.

## Author Contributions

**Promporn Piboon:** data curation (lead), formal analysis (lead), investigation (supporting), software (lead), writing – original draft (equal). **Janine Brown:** supervision (equal), writing – review and editing (lead). **Patcharaporn Kaewmong:** data curation (equal), resources (equal), supervision (equal), visualization (equal). **Kongkiat Kittiwattanawong:** data curation (equal), resources (equal), supervision (equal), visualization (equal). **Korakot Nganvongpanit:** funding acquisition (lead), methodology (equal), project administration (lead), supervision (equal), validation (equal), visualization (equal), writing – original draft (equal), writing – review and editing (equal).

## Conflicts of Interest

The authors declare no conflicts of interest.

## Supporting information


Appendix S1.

## Data Availability

Data availability in a supplementary file.

## References

[ece371014-bib-0001] Aggarwal, R. K. , A. Lalremruata , T. P. Velavan , A. Pavani Sowjanya , and L. Singh . 2008. “Development and Characterization of Ten Novel Microsatellite Markers From Olive Ridley Sea Turtle (*Lepidochelys olivacea*).” Conservation Genetics 9: 981–984.

[ece371014-bib-0002] Akkajit, P. , and A. Khongsang . 2022. “Distribution of Microplastics Along Mai Khao Coastline, Phuket.” Journal of Engineering & Technological Sciences 54: 220105.

[ece371014-bib-0003] Akkajit, P. , S. Thongnonghin , S. Sriraksa , and S. Pumsri . 2019. “Preliminary Study of Distribution and Quantity of Plastic‐Debris on Beaches Along the Coast at Phuket Province.” Applied Environmental Research 41: 54–62.

[ece371014-bib-0004] Akkajit, P. , D. Tipmanee , P. Cherdsukjai , T. Suteerasak , and S. Thongnonghin . 2021. “Occurrence and Distribution of Microplastics in Beach Sediments Along Phuket Coastline.” Marine Pollution Bulletin 169: 112496. 34023587 10.1016/j.marpolbul.2021.112496

[ece371014-bib-0005] Allard, M. W. , M. M. Miyamoto , K. A. Bjorndal , A. B. Bolten , and B. W. Bowen . 1994. “Support for Natal Homing in Green Turtles From Mitochondrial DNA Sequences.” Copeia 1994: 34–41.

[ece371014-bib-0006] Arantes, L. S. , S. M. Vargas , and F. R. Santos . 2020. “Global Phylogeography of the Critically Endangered Hawksbill Turtle (*Eretmochelys imbricata*).” Genetics and Molecular Biology 43: e20190264. 32555943 10.1590/1678-4685-GMB-2019-0264 PMC7288670

[ece371014-bib-0007] Avens, L. , J. C. Taylor , L. R. Goshe , T. T. Jones , and M. Hastings . 2009. “Use of Skeletochronological Analysis to Estimate the Age of Leatherback Sea Turtles *Dermochelys coriacea* in the Western North Atlantic.” Endangered Species Research 8: 165–177.

[ece371014-bib-0008] Bailey, H. , S. Fossette , S. J. Bograd , et al. 2012. “Movement Patterns for a Critically Endangered Species, the Leatherback Turtle (*Dermochelys coriacea*), linked to Foraging Success and Population Status.” PLoS One 7: e36401. 22615767 10.1371/journal.pone.0036401 PMC3354004

[ece371014-bib-0009] Baltazar‐Soares, M. , J. D. Klein , S. M. Correia , et al. 2020. “Distribution of Genetic Diversity Reveals Colonization Patterns and Philopatry of the Loggerhead Sea Turtles Across Geographic Scales.” Scientific Reports 10: 18001. 33093463 10.1038/s41598-020-74141-6 PMC7583243

[ece371014-bib-0010] Bass, A. L. , D. Good , K. Bjorndal , et al. 1996. “Testing Models of Female Reproductive Migratory Behaviour and Population Structure in the Caribbean Hawksbill Turtle, *Eretmochelys imbricata*, With mtDNA Sequences.” Molecular Ecology 5: 321–328. 8688954

[ece371014-bib-0011] Benson, S. R. , P. H. Dutton , C. Hitipeuw , B. Samber , J. Bakarbessy , and D. Parker . 2007. “Post‐Nesting Migrations of Leatherback Turtles (*Dermochelys coriacea*) From Jamursba‐Medi, Bird's Head Peninsula, Indonesia.” Chelonian Conservation and Biology 6: 150–154.

[ece371014-bib-0012] Boero, F. , L. Brotz , M. J. Gibbons , S. Piraino , and S. Zampardi . 2016. “3.10 Impacts and Effects of Ocean Warming on Jellyfish.” In Explaining Ocean Warming: Causes, Scale, Effects and Consequences, 213–237. International Union for Conservation of Nature.

[ece371014-bib-0013] Booth, D. T. , E. Burgess , J. McCosker , and J. M. Lanyon . 2004. “The Influence of Incubation Temperature on Post‐Hatching Fitness Characteristics of Turtles.” International Congress Series 1275: 226–233.

[ece371014-bib-0014] Bowen, B. , A. Clark , F. Abreu‐Grobois , A. Chaves , H. Reichart , and R. Ferl . 1997. “Global Phylogeography of the Ridley Sea Turtles (*Lepidochelys* Spp.) as Inferred From Mitochondrial DNA Sequences.” Genetica 101: 179–189. 9692227 10.1023/a:1018382415005

[ece371014-bib-0015] Bowen, B. W. , and S. Karl . 2007. “Population Genetics and Phylogeography of Sea Turtles.” Molecular Ecology 16: 4886–4907. 17944856 10.1111/j.1365-294X.2007.03542.x

[ece371014-bib-0016] Camacho‐Sánchez, F. Y. , J. A. Narváez‐Zapata , H. H. Acosta‐Sánchez , et al. 2022. “Molecular Identification and Novel Mitochondrial COI Gene Haplotypes of Nesting Kemp's Ridley Turtles (*Lepidochelys kempii*) in Rancho Nuevo Sanctuary, Mexico.” Diversity 14: 390.

[ece371014-bib-0017] Charles, K. E. , C. E. Morrall , J. J. Edwards , et al. 2023. “Environmental and Nesting Variables Associated With Atlantic Leatherback Sea Turtle (*Dermochelys coriacea*) Embryonic and Hatching Success Rates in Grenada, West Indies.” Animals 13: 685. 36830474 10.3390/ani13040685 PMC9951857

[ece371014-bib-0018] Choi, E. , K. E. Charles , K. L. Charles , K. M. Stewart , C. E. Morrall , and M. M. Dennis . 2020. “Leatherback Sea Turtle ( *Dermochelys coriacea* ) Embryo and Hatchling Pathology in Grenada, With Comparison to St. Kitts.” Chelonian Conservation and Biology 19: 111–123.

[ece371014-bib-0019] Chua, T. H. , and J. Furtado . 1988. “Nesting Frequency and Clutch Size in Dermochelys coriacea in Malaysia.” Journal of Herpetology 22: 208–218.

[ece371014-bib-0020] Chueasa, B. , U. W. Humphries , and M. Waqas . 2024. “Influence of El Niño Southern Oscillation on Precipitation Variability in Northeast Thailand.” MethodsX 13: 102954. 39315397 10.1016/j.mex.2024.102954 PMC11417571

[ece371014-bib-0021] Crim, J. , L. D. Spotila , J. R. Spotila , et al. 2002. “The Leatherback Turtle, *Dermochelys coriacea*, Exhibits Both Polyandry and Polygyny.” Molecular Ecology 11: 2097–2106. 12296951 10.1046/j.1365-294x.2002.01591.x

[ece371014-bib-0022] Dodge, K. L. , B. Galuardi , T. J. Miller , and M. E. Lutcavage . 2014. “Leatherback Turtle Movements, Dive Behavior, and Habitat Characteristics in Ecoregions of the Northwest Atlantic Ocean.” PLoS One 9: e91726. 24646920 10.1371/journal.pone.0091726 PMC3960146

[ece371014-bib-0023] Doyle, T. K. 2007. Leatherback Sea Turtles (Dermochelys coriacea) in Irish Waters. National Parks and Wildlife Service.

[ece371014-bib-0024] Dutton, D. L. , P. H. Dutton , M. Chaloupka , and R. H. Boulon . 2005. “Increase of a Caribbean Leatherback Turtle *Dermochelys coriacea* Nesting Population Linked to Long‐Term Nest Protection.” Biological Conservation 126: 186–194.

[ece371014-bib-0025] Dutton, P. H. , B. W. Bowen , D. W. Owens , A. Barragan , and S. K. Davis . 1999. “Global Phylogeography of the Leatherback Turtle (*Dermochelys coriacea*).” Journal of Zoology 248: 397–409.

[ece371014-bib-0026] Dutton, P. H. , C. Hitipeuw , M. Zein , et al. 2007. “Status and Genetic Structure of Nesting Populations of Leatherback Turtles (*Dermochelys coriacea*) in the Western Pacific.” Chelonian Conservation and Biology 6: 47–53.

[ece371014-bib-0027] Dutton, P. H. , S. E. Roden , K. R. Stewart , et al. 2013. “Population Stock Structure of Leatherback Turtles (*Dermochelys coriacea*) in the Atlantic Revealed Using mtDNA and Microsatellite Markers.” Conservation Genetics 14: 625–636.

[ece371014-bib-0028] Eckert, K. L. , and C. Luginbuhl . 1988. “Death of a Giant.” Marine Turtle Newsletter 43: 2–3.

[ece371014-bib-0029] Eckert, S. A. , K. L. Eckert , P. Ponganis , and G. Kooyman . 1989. “Diving and Foraging Behavior of Leatherback Sea Turtles (*Dermochelys coriacea*).” Canadian Journal of Zoology 67: 2834–2840.

[ece371014-bib-0030] Esposito, M. , S. Canzanella , D. Iaccarino , et al. 2023. “Trace Elements and Persistent Organic Pollutants in Unhatched Loggerhead Turtle Eggs From an Emerging Nesting Site Along the Southwestern Coasts of Italy, Western Mediterranean Sea.” Animals 13: 1075. 36978615 10.3390/ani13061075 PMC10044507

[ece371014-bib-0031] Ewert, M. A. , D. R. Jackson , and C. E. Nelson . 1994. “Patterns of Temperature‐Dependent Sex Determination in Turtles.” Journal of Experimental Zoology 270: 3–15.

[ece371014-bib-0032] Ferraroli, S. , J.‐Y. Georges , P. Gaspar , and Y. L. Maho . 2004. “Where Leatherback Turtles Meet Fisheries.” Nature 429: 521–522. 15175741 10.1038/429521a

[ece371014-bib-0033] FitzSimmons, N. N. , S. D. Pittard , N. McIntyre , et al. 2020. “Phylogeography, Genetic Stocks, and Conservation Implications for an Australian Endemic Marine Turtle.” Aquatic Conservation: Marine and Freshwater Ecosystems 30: 440–460.

[ece371014-bib-0034] Furler, S. 2005. “Hatching Success of the Leatherback Sea Turtle, *Dermochelys coriacea*, in Natural and Relocated Nests on Gandoca Beach, Costa Rica.” Verlag nicht ermittelbar.

[ece371014-bib-0035] Georges, J.‐Y. , and S. Fossette . 2006. “Estimating Body Mass in Leatherback Turtles *Dermochelys coriacea* .” Marine Ecology Progress Series 318: 255–262.

[ece371014-bib-0036] Girondot, M. , B. Mourrain , D. Chevallier , and M. H. Godfrey . 2021. “Maturity of a Giant: Age and Size Reaction Norm for Sexual Maturity for Atlantic Leatherback Turtles.” Marine Ecology 42: e12631.

[ece371014-bib-0037] Glen, F. , and N. Mrosovsky . 2004. “Antigua Revisited: The Impact of Climate Change on Sand and Nest Temperatures at a Hawksbill Turtle (*Eretmochelys imbricata*) Nesting Beach.” Global Change Biology 10: 2036–2045.

[ece371014-bib-0038] Godley, B. , A. Broderick , R. Frauenstein , F. Glen , and G. Hays . 2002. “Reproductive Seasonality and Sexual Dimorphism in Green Turtles.” Marine Ecology Progress Series 226: 125–133.

[ece371014-bib-0039] González‐Garza, B. I. , A. Stow , L. F. Sánchez‐Teyer , and O. Zapata‐Pérez . 2015. “Genetic Variation, Multiple Paternity, and Measures of Reproductive Success in the Critically Endangered Hawksbill Turtle (*Eretmochelys imbricata*).” Ecology and Evolution 5: 5758–5769. 26811751 10.1002/ece3.1844 PMC4717338

[ece371014-bib-0040] Hamann, M. , C. Limpus , G. Hughes , J. Mortimer , and N. Pilcher . 2006. Assessment of the Conservation Status of the Leatherback Turtle in the Indian Ocean and South East Asia, Including Consideration of the Impacts of the December 2004 Tsunami on Turtles and Turtle Habitats. IOSEA Marine Turtle MoU Secretariat.

[ece371014-bib-0041] Hays, G. C. , A. C. Broderick , B. J. Godley , P. Luschi , and W. J. Nichols . 2003. “Satellite Telemetry Suggests High Levels of Fishing‐Induced Mortality in Marine Turtles.” Marine Ecology Progress Series 262: 305–309.

[ece371014-bib-0042] Hays, G. C. , V. J. Hobson , J. D. Metcalfe , D. Righton , and D. W. Sims . 2006. “Flexible Foraging Movements of Leatherback Turtles Across the North Atlantic Ocean.” Ecology 87: 2647–2656. 17089672 10.1890/0012-9658(2006)87[2647:ffmolt]2.0.co;2

[ece371014-bib-0043] Hays, G. C. , J. D. Houghton , C. Isaacs , R. S. King , C. Lloyd , and P. Lovell . 2004a. “First Records of Oceanic Dive Profiles for Leatherback Turtles, *Dermochelys coriacea*, Indicate Behavioural Plasticity Associated With Long‐Distance Migration.” Animal Behaviour 67: 733–743.

[ece371014-bib-0044] Hays, G. C. , J. D. Houghton , and A. E. Myers . 2004b. “Pan‐Atlantic Leatherback Turtle Movements.” Nature 429: 522. 15175742 10.1038/429522a

[ece371014-bib-0045] Hendrickson, J. R. , and E. Balasingam . 1966. “Nesting Beach Preferences of Malayan Sea Turtles.” Bulletin of the National Museum Singapore 33: 69–76.

[ece371014-bib-0046] James, M. 2001. Assessment and Update Status Report on the Leatherback Turtle (Dermochelys coriacea) in Canada. Committee on the Status of Endangered Wildlife in Canada.

[ece371014-bib-0047] James, M. 2004. “ *Dermochelys coriacea* (Leatherback Sea Turtle) Penis Display.” Herpetological Review 35: 264.

[ece371014-bib-0048] James, M. C. , C. Andrea Ottensmeyer , and R. A. Myers . 2005a. “Identification of High‐Use Habitat and Threats to Leatherback Sea Turtles in Northern Waters: New Directions for Conservation.” Ecology Letters 8: 195–201.

[ece371014-bib-0049] James, M. C. , R. A. Myers , and C. A. Ottensmeyer . 2005b. “Behaviour of Leatherback Sea Turtles, *Dermochelys coriacea*, During the Migratory Cycle.” Proceedings of the Royal Society B: Biological Sciences 272: 1547–1555. 10.1098/rspb.2005.3110 PMC1559844 16048769

[ece371014-bib-0050] Janzen, F. J. 1994. “Climate Change and Temperature‐Dependent Sex Determination in Reptiles.” Proceedings of the National Academy of Sciences of the United States of America 91, no. 16: 7487–7490. 10.1073/pnas.91.16.7487. 8052608 PMC44426

[ece371014-bib-0051] Jones, T. T. , M. D. Hastings , B. L. Bostrom , D. Pauly , and D. R. Jones . 2011. “Growth of Captive Leatherback Turtles, *Dermochelys coriacea*, With Inferences on Growth in the Wild: Implications for Population Decline and Recovery.” Journal of Experimental Marine Biology and Ecology 399: 84–92.

[ece371014-bib-0052] Jonsen, I. D. , R. A. Myers , and M. C. James . 2007. “Identifying Leatherback Turtle Foraging Behaviour From Satellite Telemetry Using a Switching State‐Space Model.” Marine Ecology Progress Series 337: 255–264.

[ece371014-bib-0053] Jordão, J. C. , A. C. V. Bondioli , F. M. Guebert , B. d. Thoisy , and L. F. d. A. Toledo . 2015. “Green Turtle (*Chelonia mydas*) Genetic Diversity at Paranaguá Estuarine Complex Feeding Grounds in Brazil.” Genetics and Molecular Biology 38: 346–352. 26500439 10.1590/S1415-475738320140353 PMC4612592

[ece371014-bib-0054] Kaewmong, P. , V. Punyapornwithaya , C. Wongfu , et al. 2022. “Nest Relocation of Leatherback Turtles (*Dermochelys coriacea*) Decrease the Rate of Non‐Developed Eggs.” 10.12982/VIS.2022.022. Veterinary Integrative Sciences 20: 279–289.

[ece371014-bib-0055] Kanghae, H. , K. Thongprajukaew , P. Suraswadi , et al. 2024. “First Successful Head‐Start Program of Leatherback Sea Turtles (*Dermochelys coriacea*) in Thailand and Proposed Dietary Strategy.” Zoo Biology 43: 110–122. 37584275 10.1002/zoo.21800

[ece371014-bib-0056] Laloë, J. O. , G. Schofield , and G. C. Hays . 2024. “Climate Warming and Sea Turtle Sex Ratios Across the Globe.” Global Change Biology 30: e17004. 37961789 10.1111/gcb.17004

[ece371014-bib-0057] Lamont, M. M. , N. Moreno , F. Y. Camacho‐Sánchez , et al. 2021. “Genetic Diversity of Immature Kemp's Ridley ( *Lepidochelys kempii* ) sea Turtles From the Northern Gulf of Mexico.” Aquatic Conservation: Marine and Freshwater Ecosystems 31: 3003–3010.

[ece371014-bib-0058] Lewison, R. L. , S. A. Freeman , and L. B. Crowder . 2004. “Quantifying the Effects of Fisheries on Threatened Species: The Impact of Pelagic Longlines on Loggerhead and Leatherback Sea Turtles.” Ecology Letters 7: 221–231.

[ece371014-bib-0059] Lorne, J. K. , and M. Salmon . 2007. “Effects of Exposure to Artificial Lighting on Orientation of Hatchling Sea Turtles on the Beach and in the Ocean.” Endangered Species Research 3: 23–30.

[ece371014-bib-0060] Luschi, P. , A. Sale , R. Mencacci , G. Hughes , J. Lutjeharms , and F. Papi . 2003. “Current Transport of Leatherback Sea Turtles (*Dermochelys coriacea*) in the Ocean.” Proceedings of the Royal Society of London. Series B: Biological Sciences 270: S129–S132. 10.1098/rsbl.2003.0036 PMC1809945 14667360

[ece371014-bib-0061] Madduppa, H. , S. Bahri , A. T. Ghozali , et al. 2021. “Population Genetic Structure of Olive Ridley (*Lepidochelys olivacea*) Across Indonesian Archipelago Revealed by Mitochondrial DNA: Implication for Management.” Regional Studies in Marine Science 41: 101600.

[ece371014-bib-0062] Maison, K. 2006. “Do Turtles Move With the Beach? Beach Profiling and Possible Effects of Development on a Leatherback ( *Dermochelys coriacea* ) Nesting Beach in Grenada.” In Twenty‐Sixth Annual Symposium on Sea Turtle Biology and Conservation, 145. International Sea Turtle Society.

[ece371014-bib-0063] Mayne, B. , A. D. Tucker , O. Berry , and S. Jarman . 2020. “Lifespan Estimation in Marine Turtles Using Genomic Promoter CpG Density.” PLoS One 15: e0236888. 32735637 10.1371/journal.pone.0236888 PMC7394378

[ece371014-bib-0064] Miller, J. D. 2017. The Biology of Sea Turtles: Reproduction in Sea Turtles. CRC Press.

[ece371014-bib-0065] Nidhinarangkoon, P. , S. Ritphring , K. Kino , and T. Oki . 2023. “Shoreline Changes From Erosion and Sea Level Rise With Coastal Management in Phuket, Thailand.” Journal of Marine Science and Engineering 11: 969.

[ece371014-bib-0066] NOAA National Oceanic and Atmospheric Administration . 2024. *Leatherback turtle* National Oceanic and Atmospheric Administration.

[ece371014-bib-0067] Paladino, F. V. , and S. J. Morreale . 2001. “Sea Turtles.” In Encyclopedia of Ocean Sciences, edited by J. H. Steele . Academic Press.

[ece371014-bib-0068] Price, E. R. , B. P. Wallace , R. D. Reina , et al. 2004. “Size, Growth, and Reproductive Output of Adult Female Leatherback Turtles *Dermochelys coriacea* .” Endangered Species Research 1: 41–48.

[ece371014-bib-0069] Purcell, J. E. 2005. “Climate Effects on Formation of Jellyfish and Ctenophore Blooms: A Review.” Journal of the Marine Biological Association of the United Kingdom 85: 461–476.

[ece371014-bib-0070] Rafferty, A. R. , P. Santidrián Tomillo , J. R. Spotila , F. V. Paladino , and R. D. Reina . 2011. “Embryonic Death Is Linked to Maternal Identity in the Leatherback Turtle (*Dermochelys coriacea*).” PLoS One 6: e21038. 21695086 10.1371/journal.pone.0021038 PMC3114868

[ece371014-bib-0071] Reboul, I. , D. Booth , and U. Rusli . 2021. “Artificial and Natural Shade: Implications for Green Turtle (*Chelonia mydas*) Rookery Management.” Ocean and Coastal Management 204: 105521.

[ece371014-bib-0072] Reina, R. D. , P. A. Mayor , J. R. Spotila , R. Piedra , and F. V. Paladino . 2002. “Nesting Ecology of the Leatherback Turtle, *Dermochelys coriacea*, at Parque Nacional Marino Las Baulas, Costa Rica: 1988–1989 to 1999–2000.” Copeia 2002: 653–664.

[ece371014-bib-0073] Resources, D. o. M. a. C. Department of Marine and Coastal Resources . 2014. “The situation of nesting female leatherback turtle”.

[ece371014-bib-0074] Robinson, N. J. , S. J. Morreale , R. Nel , and F. V. Paladino . 2016. “Coastal Leatherback Turtles Reveal Conservation Hotspot.” Scientific Reports 6: 37851. 27886262 10.1038/srep37851 PMC5122952

[ece371014-bib-0075] Robinson, N. J. , and F. V. Paladino . 2013. Sea Turtles. Reference Module in Earth Systems and Environmental Sciences. Elsevier.

[ece371014-bib-0076] Roden, S. E. , K. R. Stewart , M. C. James , K. L. Dodge , F. Dell'Amico , and P. H. Dutton . 2017. “Genetic Fingerprinting Reveals Natal Origins of Male Leatherback Turtles Encountered in the Atlantic Ocean and Mediterranean Sea.” Marine Biology 164: 1–9.27980349

[ece371014-bib-0077] Saba, V. S. , J. R. Spotila , F. P. Chavez , and J. A. Musick . 2008. “Bottom‐Up and Climatic Forcing on the Worldwide Population of Leatherback Turtles.” Ecology 89: 1414–1427. 18543633 10.1890/07-0364.1

[ece371014-bib-0078] Santidrián Tomillo, P. , J. S. Suss , B. P. Wallace , et al. 2009. “Influence of Emergence Success on the Annual Reproductive Output of Leatherback Turtles.” Marine Biology 156: 2021–2031.

[ece371014-bib-0079] Santidrián Tomillo, P. , E. Vélez , R. D. Reina , R. Piedra , F. V. Paladino , and J. R. Spotila . 2007. “Reassessment of the Leatherback Turtle (*Dermochelys coriacea*) Nesting Population at Parque Nacional Marino Las Baulas, Costa Rica: Effects of Conservation Efforts.” Chelonian Conservation and Biology 6: 54–62.

[ece371014-bib-0080] Santidrián‐Tomillo, P. , N. J. Robinson , L. G. Fonseca , et al. 2017. “Secondary Nesting Beaches for Leatherback Turtles on the Pacific Coast of Costa Rica.” Latin American Journal of Aquatic Research 45: 563–571.

[ece371014-bib-0081] Sarti, M. , S. A. Eckert , N. Garcia , and A. R. Barragan . 1996. “Decline of the world's Largest Nesting Assemblage of Leatherback Turtles.” Marine Turtle Newsletter 74: 2–5.

[ece371014-bib-0082] Sato, C. L. 2017. Peribodic Status Review for the Leatherback Sea Turtle in Washington. Washington Department of Fish and Wildlife.

[ece371014-bib-0083] Segniagbeto, G. , D. Okangny , A. Mondedji , et al. 2017. “Sea Turtle Bycatch Analysis Revealed That Site Influenced Mortality More Than Net Types Along the Coast of Togo.” Vie et Milieu 67: 227–234.

[ece371014-bib-0084] Simantiris, N. 2024. “The Impact of Climate Change on Sea Turtles: Current Knowledge, Scientometrics, and Mitigation Strategies.” Science of the Total Environment 932: 171354. 10.1016/j.scitotenv.2024.171354 38460688

[ece371014-bib-0085] Somphong, C. , K. Udo , S. Ritphring , and H. Shirakawa . 2022. “An Estimate of the Value of the Beachfront With Respect to the Hotel Room Rates in Thailand.” Ocean and Coastal Management 226: 106272.

[ece371014-bib-0086] Sönmez, B. , D. Sammy , Ş. Yalçın‐Özdilek , et al. 2008. “A Stranded Leatherback Sea Turtle in the Northeastern Mediterranean, Hatay, Turkey.” Marine Turtle Newsletter 119: 12–13.

[ece371014-bib-0087] Spotila, J. R. , R. D. Reina , A. C. Steyermark , P. T. Plotkin , and F. V. Paladino . 2000. “Pacific Leatherback Turtles Face Extinction.” Nature 405: 529–530. 10850701 10.1038/35014729

[ece371014-bib-0088] Spotila, J. R. , and P. S. Tomillo . 2015. The Leatherback Turtle: Biology and Conservation. JHU Press.

[ece371014-bib-0089] Svarachorn, T. , A. J. Temple , and P. Berggren . 2023. “Marine Megafauna Catch in Thai Small‐Scale Fisheries.” Aquatic Conservation: Marine and Freshwater Ecosystems 33: 1245–1262.

[ece371014-bib-0090] Swimmer, Y. , A. Gutierrez , K. Bigelow , et al. 2017. “Sea Turtle Bycatch Mitigation in US Longline Fisheries.” Frontiers in Marine Science 4: 260.

[ece371014-bib-0091] Tanabe, L. K. , J. E. Cochran , R. S. Hardenstine , K. Scott , and M. L. Berumen . 2023. “A Preliminary Report of Plastic Ingestion by Hawksbill and Green Turtles in the Saudi Arabian Red Sea.” Animals 13: 314. 36670854 10.3390/ani13020314 PMC9854423

[ece371014-bib-0092] Thums, M. , S. D. Whiting , J. Reisser , et al. 2016. “Artificial Light on Water Attracts Turtle Hatchlings During Their Near Shore Transit.” Royal Society Open Science 3: 160142. 27293795 10.1098/rsos.160142 PMC4892457

[ece371014-bib-0093] Vargas, S. M. , F. C. Araújo , D. S. Monteiro , et al. 2008. “Genetic Diversity and Origin of Leatherback Turtles ( *Dermochelys coriacea* ) From the Brazilian Coast.” Journal of Heredity 99: 215–220. 18252731 10.1093/jhered/esm120

[ece371014-bib-0094] Vargas, S. M. , A. C. Barcelos , R. G. Rocha , et al. 2022. “Genetic Monitoring of the Critically Endangered Leatherback Turtle (*Dermochelys coriacea*) in the South West Atlantic.” Regional Studies in Marine Science 55: 102530.

[ece371014-bib-0095] Vargas, S. M. , M. P. Jensen , S. Y. Ho , et al. 2016. “Phylogeography, Genetic Diversity, and Management Units of Hawksbill Turtles in the Indo‐Pacific.” Journal of Heredity 107: 199–213. 26615184 10.1093/jhered/esv091 PMC4885228

[ece371014-bib-0096] Vélez‐Rubio, G. M. , L. Prosdocimi , M. López‐Mendilaharsu , et al. 2023. “Natal Origin and Spatiotemporal Distribution of Leatherback Turtle (*Dermochelys coriacea*) Strandings at a Foraging Hotspot in Temperate Waters of the Southwest Atlantic Ocean.” Animals 13: 1285. 37106848 10.3390/ani13081285 PMC10134985

[ece371014-bib-0097] Wallace, B. P. , A. D. DiMatteo , B. J. Hurley , et al. 2010. “Regional Management Units for Marine Turtles: A Novel Framework for Prioritizing Conservation and Research Across Multiple Scales.” PLoS One 5: e15465. 21253007 10.1371/journal.pone.0015465 PMC3003737

[ece371014-bib-0098] Wallace, B. P. , M. Tiwari , and M. Girondot . 2013. “Dermochelys Coriacea. The IUCN Red List of Threatened Species 2013: E.T6494A43526147.” 10.2305/IUCN.UK.2013-2.RLTS.T6494A43526147.en.

[ece371014-bib-0099] Wilcox, C. , M. Puckridge , Q. A. Schuyler , K. Townsend , and B. D. Hardesty . 2018. “A Quantitative Analysis Linking Sea Turtle Mortality and Plastic Debris Ingestion.” Scientific Reports 8: 12–536. 30213956 10.1038/s41598-018-30038-z PMC6137038

[ece371014-bib-0100] Witt, M. J. , A. C. Broderick , D. J. Johns , et al. 2007. “Prey Landscapes Help Identify Potential Foraging Habitats for Leatherback Turtles in the NE Atlantic.” Marine Ecology Progress Series 337: 231–243.

[ece371014-bib-0101] Wongfu, C. , W. Prasitwiset , A. Poommouang , et al. 2022. “Genetic Diversity in Leatherback Turtles (*Dermochelys coriacea*) Along the Andaman Sea of Thailand.” Diversity 14: 764.

[ece371014-bib-0102] Wyneken, J. , K. J. Lohmann , and J. A. Musick . 2013. The Biology of Sea Turtles. CRC press.

[ece371014-bib-0103] Yao, Y.‐T. , Y. Du , J.‐X. Pan , C.‐X. Lin , X. Ji , and W.‐H. You . 2022. “Incubating Green Turtle (*Chelonia mydas*) Eggs at Constant Temperatures: Hatching Success, Hatchling Morphology and Post‐Hatch Growth.” Journal of Thermal Biology 104: 103182. 35180961 10.1016/j.jtherbio.2021.103182

[ece371014-bib-0104] Yoshikawa, N. , N. Kamezaki , I. Kawazu , S. Hirai , and S. Taguchi . 2016. “Stock Origin of the Leatherback Turtles (*Dermochelys coriacea*) Found in the Vicinity of Japan Revealed by mtDNA Haplotypes.” Current Herpetology 35: 115–121.

